# Establishment of a Genetic Transformation System for *Hippophae gyantsensis* and the Regulatory Role of *Hgfw2.2* and *Hgfw3.2* in Fruit Size

**DOI:** 10.3390/plants15111615

**Published:** 2026-05-25

**Authors:** Yaqing Zhang, Yumeng Gao, Chunxia Chen, Anqi Zhao, Yunhua Wu, Lisha Shi, Qixuan Wei, Zijie Zhou, Xiaoming Yang, Meiling Ming, Lin Zhang, Fuliang Cao, Fangfang Fu

**Affiliations:** 1 State Key Laboratory of Tree Genetics and Breeding, Co-Innovation Center for Sustainable Forestry in Southern China, Nanjing Forestry University, Nanjing 210037, China; zhangyaqing@njfu.edu.cn (Y.Z.); yumenggao@njfu.edu.cn (Y.G.); chunxiachen@njfu.edu.cn (C.C.); anqi@njfu.edu.cn (A.Z.); sls@njfu.edu.cn (L.S.); weiqixuan@njfu.edu.cn (Q.W.); zijiezhou08@outlook.com (Z.Z.); xmyang@njfu.edu.cn (X.Y.); mingmeiling@njfu.edu.cn (M.M.); flcao@njfu.edu.cn (F.C.); 2 Key Laboratory of Cultivation and Protection for Non-Wood Forest Trees of the Ministry of Education, Central South University of Forestry and Technology, Changsha 410004, China; 15289005289@163.com (Y.W.); triwoodtim918@126.com (L.Z.); 3 Forestry Investigation and Planning Research Institute of Tibet Autonomous Region, Lhasa 850000, China

**Keywords:** *Hippophae gyantsensis*, genetic transformation, fruit size, Tibet

## Abstract

*Hippophae gyantsensis* Lian is an important native tree species in the “One River, Two Streams” valley of Tibet, valued for its ecological restoration potential and nutrient-rich fruits. However, this species has several limitations, including a long fruiting cycle (3–5 years to flowering and 10–15 years to reach peak fruit production), small fruit size, and numerous branch thorns. These traits hinder large-scale cultivation and mechanized harvesting, creating an urgent need for improved varieties with larger fruit and higher yield. In this study, we established an efficient *Agrobacterium*-mediated genetic transformation system for *H. gyantsensis* using hypocotyls as explants. Under optimized conditions (OD_600_ = 0.5, AS = 200 μmol/L, infection time = 15 min), the transformation efficiency reached 36.67% (calculated as the number of PCR-positive plants divided by the total number of explants initially inoculated with *Agrobacterium*). A rooting rate of 12.5% was achieved using 100 mg/L rooting powder (ABT1) for 40 min, resulting in an overall success rate of approximately 4–5%. Furthermore, we identified and cloned two fruit-size-related genes, *Hgfw2.2* and *Hgfw3.2*, from *H. gyantsensis*. Heterologous expression of *Hgfw2.2* and *Hgfw3.2* in tomato decreased and increased fruit size, respectively, consistent with their regulatory roles in fruit development. Given the positive regulatory effect of *Hgfw3.2*, this gene was further transformed into *H. gyantsensis*. This study represents the first report of a stable genetic transformation platform for *H. gyantsensis*, providing a robust technical foundation for future molecular breeding and the development of improved, large-fruited varieties.

## 1. Introduction

Sea buckthorn belongs to the genus *Hippophae* within the family Elaeagnaceae and is also commonly known as sour thorn, vinegar willow, or black thorn. It is a deciduous shrub or small tree. China possesses the richest natural *Hippophae* germplasm resources in the world, with its cultivated area accounting for 93% of the global total, ranking first internationally. *Hippophae gyantsensis* Lian is the most prevalent sea buckthorn species in Tibet, mainly distributed in gravelly riverbeds and floodplains of the Yarlung Zangbo River Basin at elevations ranging from 3500 to 5000 m [1,2,3]. This species is primarily propagated by seeds, while rapid propagation can be achieved through asexual reproduction techniques such as grafting, cuttings, and tissue culture. Owing to its fast growth rate and strong asexual reproductive capacity, *H. gyantsensis* plays a key role in ecological restoration, vegetation reconstruction, and windbreak and sand fixation.

In addition, *Hippophae* is abundant in flavonoids, superoxide dismutase (SOD), and other bioactive components, which confer significant antioxidant, anti-inflammatory, and immunomodulatory properties [4,5,6]. Through precise extraction, separation, and purification technologies, sea buckthorn-derived pharmaceuticals and functional foods can be effectively developed. All these attributes indicate that *H. gyantsensis* has significant research and breeding value.

As a biotechnological approach, transgenic technology breaks the species barriers of traditional biological breeding, enables the recombination of genetic material between different species, and significantly enhances breeding efficiency. With the advancement of modern technology, various plant genetic transformation techniques have emerged, including *Agrobacterium*-mediated transformation, virus-mediated transformation, pollen tube pathway method, gene gun method, protoplast-mediated transformation, microinjection, electroporation, and polyethylene glycol (PEG)-mediated transformation [7,8]. Among these, the *Agrobacterium*-mediated method is the most widely used in plant breeding and molecular biology research. Owing to *Agrobacterium*’s inherent ability to transfer genetic material into host cells, this method features simple operation, low cost, and high transformation efficiency [9].

At present, research on the genetic transformation of *Hippophae* (sea buckthorn) is still in the preliminary exploration stage, with relatively few relevant literature reports. Regarding the optimization of the *Agrobacterium*-mediated genetic transformation system, a study systematically analyzed the key transformation parameters of *Hippophae rhamnoides* subsp. *sinensis* Rousi, including explant type, pre-culture duration, acetosyringone concentration, infection time, and co-culture duration, and finally achieved a positive transformation rate of 6.9%. Currently, our research group has established a tissue culture regeneration system for *H. gyantsensis* using cotyledons and hypocotyls as explants [10], whereas studies on its genetic transformation have not yet been reported in the literature.

Fruits are vital sources of human nutrition, with size significantly impacting both commercial value and nutrient accumulation [11,12]. *fw2.2* and *fw3.2* are two landmark genes regulating fruit weight. *fw2.2*, the first identified fruit size QTL, negatively regulates cell division. Its protein contains a conserved PLAC8 motif present across kingdoms [13,14]. Orthologs such as maize *CNR* and soybean *GmFWL1* demonstrate its functional conservation: *CNR* overexpression inhibits growth by restricting cell number, while its silencing promotes organ enlargement [15]. In addition, *GmFWL1* plays additional roles in symbiotic nitrogen fixation [16]. Thus, *fw2.2* consistently acts as a negative regulator of cell division across diverse species.

Conversely, *fw3.2* acts as a positive regulator, with *KLUH* (a member of the CYP78A subfamily) identified as the primary candidate gene. Its homolog, *AtKLUH*, regulates organ size in *Arabidopsis* by promoting cell proliferation [17]. In tomatoes, *fw3.2* increases fruit weight by enhancing pericarp cell numbers and delaying ripening. In *Arabidopsis thaliana*, members such as *AtCYP78A5*, *AtCYP78A6*, and *AtCYP78A9* are all closely associated with seed size regulation [18,19]. In soybeans, *GmCYP78A10* functions in fruit/seed enlargement [20]. Additionally, overexpression of *GmCYP78A72* in both *Arabidopsis* and soybeans results in the enlargement of organs such as seeds, petals, and carpels [21]. In wheat, *TaCYP78A3* significantly increases seed weight by promoting seed coat cell proliferation, while its silencing leads to seed shrinkage [22]. Collectively, *fw3.2* and the broader *CYP78A* family function as positive regulators of fruit and seed size across various crops.

In the breeding of *H. gyantsensis*, fruit size is a key trait for increasing yield and thus holds significant breeding value. Based on the tissue culture and regeneration system previously established in our laboratory [10], this study successfully developed a stable genetic transformation system for *H*. *gyantsensis*. Meanwhile, the *FWL* and *CYP78A* gene families associated with fruit size were systematically identified; among these families, the *Hgfw2.2* and *Hgfw3.2* genes were heterologously transformed into tomato, which were consistent with the hypothesis that *Hgfw2.2* and *Hgfw3.2* influence organ size potentially through cell number modulation. Because the *Hgfw3.2* gene has a positive regulatory effect, the researchers further introduced it into *H. gyantsensis* using genetic transformation techniques. Collectively, this work provides a solid theoretical basis and reliable technical support for the genetic improvement of large-fruited varieties of *H. gyantsensis*.

## 2. Results

### 2.1. Screening of the Optimal Antibiotic Concentration for Hippophae gyantsensis Lian

**Figure 1:**
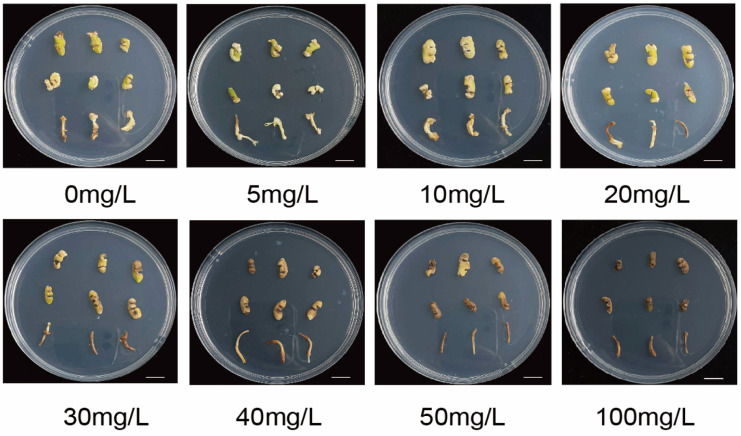
Screening for optimal antibiotic concentration. Untransformed cotyledons and hypocotyls of *H. gyantsensis* were cultured on media containing 0, 5, 10, 20, 30, 40, 50, and 100 mg/L kanamycin for 30 days to observe the growth status. In each plate, the top six were the cotyledons, and the bottom three were the hypocotyls from the same plant. Scale bar = 1 cm.

As no genetic transformation system for *H. gyantsensis* has been previously reported, we first optimized the antibiotic concentrations in the post-transformation screening medium. To this end, untransformed cotyledons and hypocotyls were inoculated on media containing various concentrations of kanamycin (0, 5, 10, 20, 30, 40, 50, and 100 mg/L), and callus induction was observed. After one month of culture, both cotyledons and hypocotyls produced calli normally on media with 0, 5, or 10 mg/L kanamycin. At 20 mg/L, some cotyledons and hypocotyls still formed calli, although hypocotyls exhibited severe browning. At 30 mg/L, cotyledons still generated a small amount of calli, whereas all hypocotyls died. At 40, 50, and 100 mg/L, neither cotyledons nor hypocotyls induced calli; instead, they gradually turned brown and died (Figure 1).

Therefore, 30 mg/L kanamycin was selected as the screening concentration for cotyledons, and 20 mg/L for hypocotyls in this experiment.

### 2.2. Agrobacteria Mediated Transformation of H. gyantsensis

**Figure 2:**
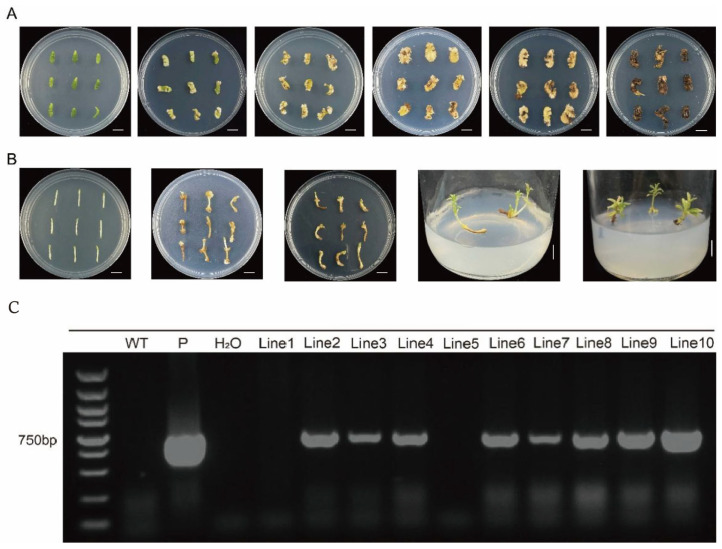
Genetic transformation process of different explants in *H. gyantsensis* and DNA identification. (**A**) Cotyledon genetic transformation process. (**B**) Hypocotyl genetic transformation process. (**C**) DNA identification of pSAK277 transgenic plants. Marker: DL5000 bp. WT represents wild-type; P and H_2_O represent plasmid and water, respectively; and Line1-10 represent transgenic plants 1–10. Scale bar = 1 cm.

Transformation and regeneration assays revealed that cotyledons could form callus under inductive culture conditions but failed to efficiently differentiate into adventitious shoots on media supplemented with selective agents, indicating their weak regenerative capacity. In contrast, hypocotyl explants not only induced callus on selective media containing resistance agents but also efficiently differentiated into robust adventitious shoots on differentiation media (Figure 2A,B), demonstrating superior regeneration potential and selection tolerance. Therefore, hypocotyls were selected as the primary recipient material for genetic transformation in this study to enhance transformation efficiency and the regeneration success of transgenic plants.

To confirm the stable integration of the target vector into the *H. gyantsensis* genome, ten regenerated plantlets were subjected to molecular detection. PCR amplification yielded the expected 731 bp target band (Primer ID: pSAK277-JD, Table S1) in eight of these plants, corresponding to a positive rate of 80% and indicating successful genomic integration of the vector. No amplification products were detected in wild-type plants (negative control) or ddH_2_O (blank control), further validating the reliability of the experimental results and the specificity of the amplified band (Figure 2C). These results collectively show that the stable genetic transformation system established for *H. gyantsensis* is reliable and functional.

### 2.3. The Effect of Different Transformation Conditions on Transformation Efficiency of H. gyantsensis

To systematically optimize key transformation parameters—namely, *Agrobacterium* strain concentration (OD_600_), acetosyringone (AS) concentration, and infection time—an orthogonal experimental design was employed (Table S2). Three factors were evaluated for their effects on transformation efficiency using range analysis (R-value): bacterial suspension concentration (A), AS concentration (B), and infiltration time (C). The optimal transformation conditions were determined based on transformation efficiency and positive rate.

As shown in Table S3, the factors affecting transformation efficiency ranked as C > B > A (infiltration time > AS concentration > bacterial suspension concentration), indicating that infiltration time was the primary influencing factor. For the positive rate, the order was A > C > B (bacterial suspension concentration > infiltration time > AS concentration), suggesting that bacterial suspension concentration played a dominant role in increasing the positive rate.

Comprehensive analysis of transformation efficiency and positive rate (Table S2) identified the optimal transformation conditions as A_2_B_3_C_2_: bacterial suspension concentration OD_600_ = 0.5, AS concentration = 200 μmol/L, and infiltration time = 15 min. Under these conditions, the highest transformation efficiency reached 36.67% (T17).

Additionally, when the bacterial suspension concentration was OD_600_ = 0.3, AS concentration was set to 100, 150, or 200 μmol/L, and infiltration time was 15 or 20 min, the positive rate reached 100%. This indicates that an appropriate combination of bacterial suspension concentration and infiltration time can effectively improve the transformation positive rate.

### 2.4. Screening for the Optimal Genetic Transformation System

**Figure 3:**
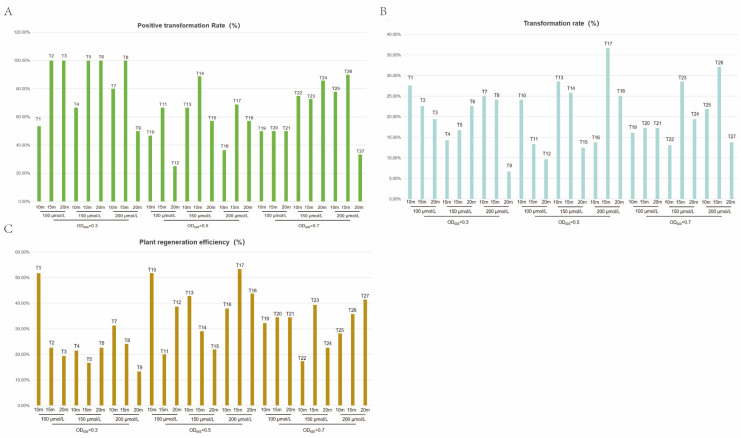
Plant regeneration efficiency, PCR-positive transformation efficiency and transformation efficiency in orthogonal experimental design. (**A**) PCR-positive transformation Rate (%) = (Number of PCR-positive plants/Number of regenerated shoots) × 100%. (**B**) Transformation Rate (%) = (Number of PCR-positive plants/Total number of explants) × 100%. (**C**) Plant regeneration efficiency (%) = (Number of regenerated shoots/Total number of explants) × 100%.

Figure 3 presents the plant regeneration efficiency, PCR-positive transformation efficiency, and overall transformation efficiency obtained from the orthogonal experimental design.

Differences were observed between the genetic transformation efficiency and the PCR-positive rate of *H. gyantsensis* (Figure 3A,B). To further optimize the genetic transformation system, transformation efficiency was adopted as the primary evaluation criterion in this study (Figure 3B). The results showed that the highest transformation efficiency was achieved under treatment T17 (OD_600_ = 0.5, AS = 200 μmol/L, infiltration time = 15 min). Therefore, this combination was determined as the optimal condition for the genetic transformation of *H. gyantsensis*.

### 2.5. Optimization of Rooting Culture Conditions for Transgenic Shoots

Some plants experience difficulties in rooting during tissue culture, which negatively affects their genetic transformation efficiency and application prospects. *H. gyantsensis* exhibits weak rooting capacity following *Agrobacterium* infection, making this a key challenge in establishing a stable genetic transformation system for this species.

**Figure 4:**
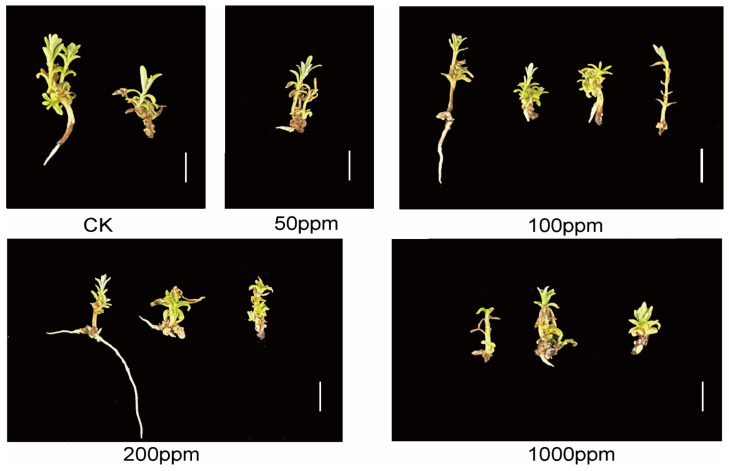
Effects of different treatments on rooting of *H. gyantsensis*. CK as a control, with no hormone treatment—ppm, part per million; 50 ppm, 100 ppm, 200 ppm, and 1000 ppm represent hormone concentrations of 50, 100, 200 and 1000 mg/L, respectively. Scale bar = 1 cm.

Therefore, when the putative positive transgenic plants reached a height of 1–2 cm, healthy shoots were excised from the shoot bases and subjected to different rooting treatments before transplantation into nutrient soil (Table S4). Rooting status was regularly observed and recorded. Thirty days later, the rooting performance of each treatment is shown in Figure 4, and the rooting rates are summarized in Table 1. Significant differences in rooting rates were observed among treatments. The results indicated that, in this study, treatment with 100 mg/L ABT1 rooting powder for 40 min exhibited the highest rooting induction efficiency, achieving a rooting rate of 12.5%.

### 2.6. Identification of Fruit-Size-Related Gene Hgfw2.2 and Hgfw3.2 in H. gyantsensis

**Figure 5:**
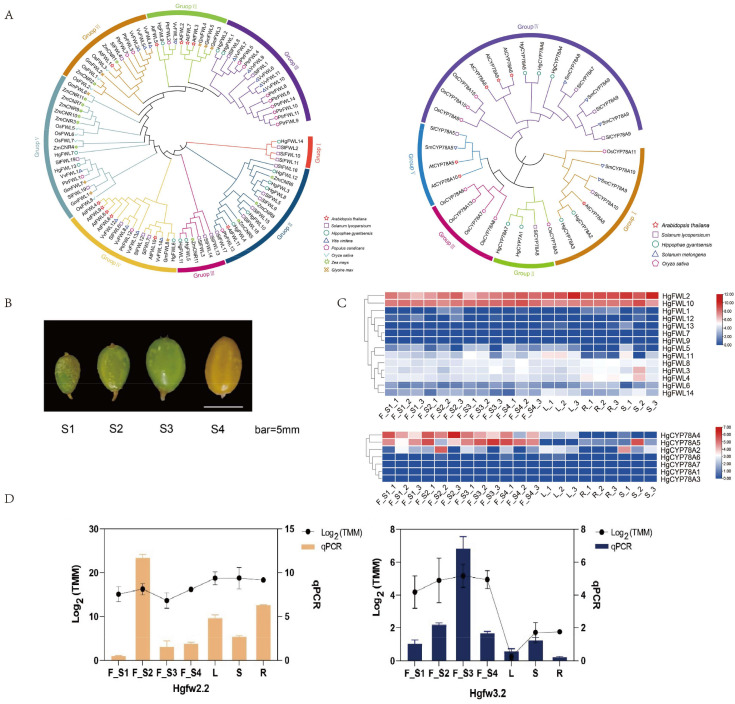
(**A**) Phylogenetic analysis of the *HgFWL* and *HgCYP78A* family. (**B**) Developmental stages of *H. gyantsensis* fruit used in this study. S1-S4 represent different sampling time points: 7.11,7.22,8.1,8.8. Scale bar = 5 mm. (**C**) Expression patterns of *HgFWL* and *HgCYP78A* in different tissues. (**D**) qRT-PCR validation of *Hgfw2.2* and *Hgfw3.2* expression. F_S1-F_S4: Fruit at different developmental stages; L: Leaf; R: Root; S: Stem. Log2(TMM) represents gene expression levels from RNA-seq; qPCR validates gene expression by fluorescence quantification.

In the breeding of *H. gyantsensis*, fruit size is a key trait for increasing yield and thus holds significant breeding value. Based on an optimized and stable genetic transformation system for this species, we have initiated molecular breeding research focusing on genes that regulate fruit size in this unique germplasm resource. The genes *fw2.2* and *fw3.2* are the first two reported to regulate fruit or seed size in many species, belonging to the FWL and CYP78A families, respectively. Both *HgFWL* and *HgCYP78A* are involved in organ size regulation. To identify *Hgfw2.2* and *Hgfw3.2*, we systematically analyzed these two gene families in *H. gyantsensis* (Figure 5A).

A total of 14 genes were identified in the *HgFWL* gene family (Table S5), which controls cell number. Their expression patterns were relatively consistent: some genes were highly expressed in fruits at various developmental stages as well as in vegetative organs (roots, stems, and leaves), whereas others showed low or undetectable expression. For example, *HgFWL2* and *HgFWL10* were stably and highly expressed throughout fruit development and were also significantly upregulated in roots, stems, and leaves (Figure 5C).

Seven genes were identified in the *HgCYP78A* gene family (Table S6), which exhibited strong tissue specificity. These genes were predominantly highly expressed in fruits but weakly expressed in roots, stems, and leaves. Specifically, in vegetative organs, *HgCYP78A2* was mainly expressed in leaves and stems, with almost no expression in roots, and its transcript abundance gradually declined during fruit development. By contrast, *HgCYP78A5* was highly expressed in stems and roots but barely detectable in leaves, and remained stably high throughout all fruit developmental stages.

To investigate the association between the *HgFWL* and *HgCYP78A* gene families and fruit size in *H. gyantsensis*, and given that most of the identified genes are homologous to their counterparts in the model plant tomato and exhibit high similarity (Figures S1 and S2), Based on the phyloanalysis tree (Figure S3), the *FWL* gene showing the highest homology to tomato *fw2.2* was selected and designated as *Hgfw2.2* (*HgFWL2*). For the *CYP78A* family, the two genes most homologous to tomato *fw3.2* (*HgCYP78A7* and *HgCYP78A1*) exhibited almost no detectable expression in the examined tissues (Figure 5C). Therefore, the gene with the next highest homology, *HgCYP78A5*, was selected and named *Hgfw3.2.* qRT-PCR results revealed that the relative expression levels of these two genes were highly consistent with the expression trends obtained from transcriptome sequencing (Figure 5D).

### 2.7. Heterologous Transformation of Hgfw2.2 and Hgfw3.2 in Tomato

**Figure 6:**
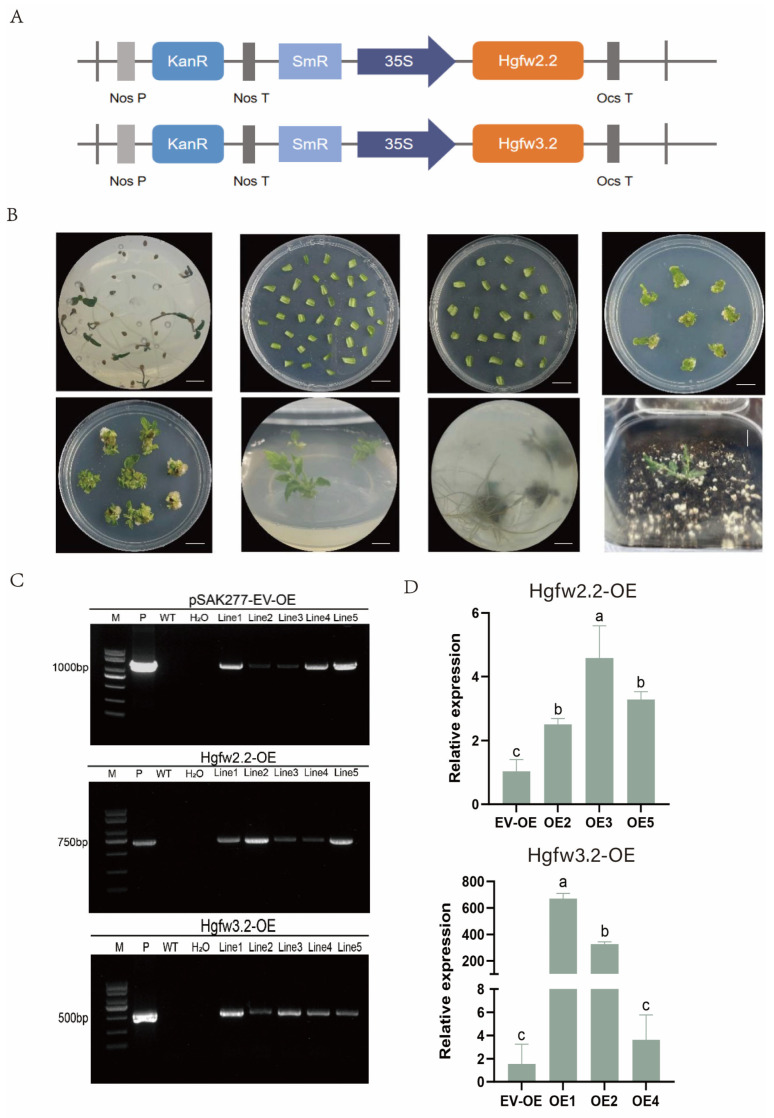
Gene expression analysis of *Hgfw2.2* and *Hgfw3.2* transgenic tomatoes. (**A**) Construction of *Hgfw2.2* and *Hgfw3.2* into the pSAK277 vector. (**B**) *Solanum lycopersicum* L. genetic transformation process. Scale bar = 1 cm. (**C**) DNA identification of *Hgfw2.2* and *Hgfw3.2* transgenic *Solanum lycopersicum* L. Marker: DL2000 bp. (**D**) Quantitative analysis of transgene expression levels in transgenic plants. Marker: DL2000 DNA ladder. EV-OE: Overexpression of Empty vector control; OE-2/OE-3/OE-5: three independent overexpression transgenic lines of *Hgfw2.2*. OE-1/OE-2/OE-4: three independent overexpression transgenic lines of *Hgfw3.2*. Data are presented as Mean ± SD (n = 3). Different lowercase letters within the same column indicate significant differences (*p* < 0.05) based on one-way ANOVA followed by Dunnett’s post hoc test.

To functionally characterize *Hgfw2.2* and *Hgfw3.2*, the full-length coding sequences of these two genes were cloned and inserted into the linearized pSAK277 vector to generate the recombinant plasmids pSAK277-*Hgfw2.2* and pSAK277-*Hgfw3.2* (Figure 6A).

The recombinant constructs were transformed into *Escherichia coli*, and after successful sequence verification, they were introduced into *Agrobacterium tumefaciens*. Heterologous transformation was then performed to investigate the functions of these fruit size-related genes. Using cotyledons of the tomato cultivar *Solanum lycopersicum* L. as explants, the recombinant plasmids pSAK277-*Hgfw2.2* and pSAK277-*Hgfw3.2* were introduced into tomato via *Agrobacterium tumefaciens*-mediated transformation (Figure 6B).

qRT-PCR analysis of the putative transgenic plants revealed that the relative expression levels of *Hgfw2.2* and *Hgfw3.2* in the transgenic lines were more than twofold higher than those in the empty vector control (EV-OE) plants (Figure 6D), confirming the successful generation of positive transgenic tomato plants overexpressing *Hgfw2.2* and *Hgfw3.2*.

### 2.8. Hgfw2.2 and Hgfw3.2 Regulate the Fruit Size of Tomato

**Figure 7:**
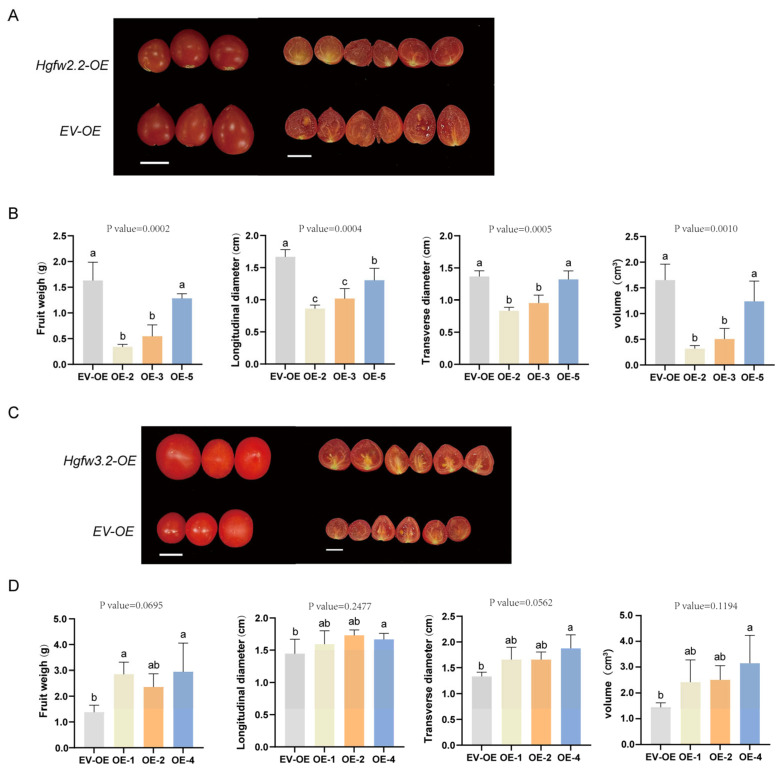
Fruit shape index analysis of *Hgfw2.2* and *Hgfw3.2* transgenic tomato plants. (**A**) Fruits of *Hgfw2.2* transgenic and control plants. (**B**) Fruit weight, fruit longitudinal diameter, fruit transverse diameter and fruit volume of *Hgfw2.2* transgenic and control plants; Different colors represent different overexpressed transgenic lines; EV-OE: overexpression of Empty vector control; OE-2/OE-3/OE-5: three independent overexpression transgenic lines of *Hgfw2.2*. Three fruits for each line were measured. (**C**) Fruits of *Hgfw3.2* transgenic and control plants. (**D**) Fruit weight, fruit longitudinal diameter, fruit transverse diameter and fruit volume of *Hgfw3.2* transgenic and control plants; Fruit volume of transgenic and control plants; Different colors represent different overexpressed transgenic lines; EV-OE: overexpression of Empty vector control; OE-1/OE-2/OE-4: three independent overexpression transgenic lines of *Hgfw3.2*. Three fruits for each line were measured. *p* values were calculated with one-way ANOVA and Dunnett’s test, n = 3. Data were presented as Mean ± SD. Scale bar = 1 cm. Different lowercase letters indicate significant differences between treatments (n = 3).

Phenotypic analysis of *Hgfw2.2*-overexpressing transgenic tomato fruits (Figure 7A,B) revealed that, compared with the empty vector control (EV-OE), fruit weight, length, and diameter were significantly reduced in all overexpression lines (OE 2, OE 3, and OE 5). Furthermore, visual inspection showed that *Hgfw2.2*-overexpressing plants produced fewer seeds or were completely seedless relative to the control plants. These results suggest that *Hgfw2.2* may function as a negative regulator of fruit size.

In contrast, phenotypic analysis of fruits from *Hgfw3.2*-overexpressing transgenic tomatoes (Figure 7C,D) showed that, compared with the empty vector control (EV-OE), all transgenic lines (OE-1, OE-2, OE-4) exhibited higher fruit weight, length, and diameter. Specifically, fruit weight in OE-1 and OE-4 increased significantly by nearly twofold, whereas OE-2 showed only a non-significant upward trend. Regarding fruit length, no significant difference was detected between OE-1 and OE-2. Notably, fruit volume and weight displayed consistent trends: the volume and weight of OE-1 and OE-4 lines were significantly higher than those of the control group, while OE-2 did not show significant differences. Therefore, *Hgfw3.2* likely plays a positive regulatory role in fruit size.

### 2.9. Homologous Transformation of Hgfw3.2 in H. gyantsensis

**Figure 8:**
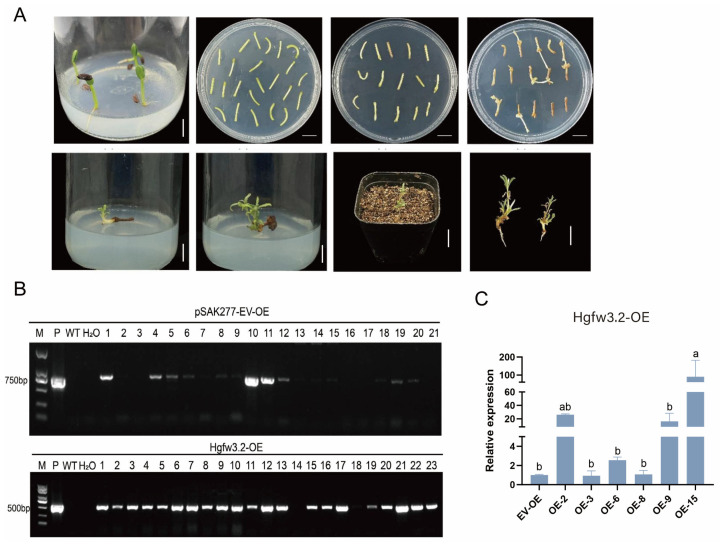
Conversion of *H. gyantsensis*. (**A**) Genetic transformation process of *H. gyantsensis*. Scale bar = 1 cm. (**B**) Molecular identification of *Hgfw3.2* transgenic *H. gyantsensis* DNA. Marker: DL2000 bp. (**C**) Quantitative analysis of transgene expression levels. Marker: DL2000 DNA ladder. EV-OE: Overexpression of Empty vector control; OE-2/OE-3/OE-6/OE-8/OE-9/OE-15: six randomly independent overexpression transgenic lines of *Hgfw3.2*. Data are presented as Mean ± SD (n = 3). Different lowercase letters within the same column indicate significant differences (*p* < 0.05) based on one-way ANOVA followed by Dunnett’s post hoc test.

Given the positive regulatory effect of *Hgfw3.2*, this gene was further introduced into *H. gyantsensis* via *Agrobacterium*-mediated transformation (Figure 8A). To verify the transformation efficiency, genomic DNA was extracted from the obtained resistant plantlets, and the target band was detected by PCR amplification (Figure 8B). Among the resistant plantlets showing the presence of the target band by PCR, six randomly selected plants were used for RNA extraction, followed by quantitative real-time PCR (qRT-PCR) analysis. The results showed that at least two of the resistant plantlets exhibited significantly higher expression levels of the *Hgfw3.2* gene compared with the control group, with expression levels exceeding those of the control by more than twofold (Figure 8C).

These findings confirm that transgenic *H*. *gyantsensis* plants overexpressing the *Hgfw3.2* gene were successfully obtained, providing reliable experimental material for subsequent studies.

## 3. Discussion

Plant genetic transformation is defined as the process by which target genes or DNA fragments are integrated into the genome of recipient plants through molecular biological approaches, resulting in stable expression and heritable transmission of the introduced sequences [23,24]. However, systematic studies on the genetic transformation system of *H. gyantsensis*, a plateau-endemic species, remain scarce to date.

Optimizing antibiotic concentration is critical for establishing plant genetic transformation systems [25]. The optimal concentration should effectively suppress non-transformed tissues while minimizing somatic cell death, as dead cells may release toxic substances that hinder the regeneration of transformed cells. Kanamycin (Kan), a commonly used selective agent, inhibits peptide synthesis in *E. coli* by blocking translocation [26]. In this study, using the kanamycin resistance gene carried by the vector, we systematically evaluated the Kan sensitivity of different *H. gyantsensis* tissues. Our results (Figure 1) showed that 20 mg/L Kan sufficiently inhibited hypocotyl growth, causing browning and necrosis, while 30 mg/L markedly suppressed cotyledon growth. Compared with other woody plants reported [27], *H. gyantsensis* exhibited higher Kan sensitivity, possibly due to its unique physiological characteristics or tissue-specific responses. These findings provide a solid foundation for establishing an efficient genetic transformation system.

Common explants for genetic transformation include leaves, stem segments, shoot tips, hypocotyls, roots, and callus [28]. In this study, cotyledons and hypocotyls from sterile *H. gyantsensis* seedlings were used as explants for *Agrobacterium*-mediated transformation to determine the optimal recipient material. The results showed that hypocotyls achieved a transformation efficiency (PCR-positive rate) of 80%. In contrast, although cotyledons formed callus after *Agrobacterium* infection, they failed to regenerate shoots and eventually turned brown and necrotic. Based on a comprehensive assessment of transformation efficiency and regenerative capacity, hypocotyls were identified as the optimal explant for establishing a genetic transformation system for *H. gyantsensis*.

Pre-culturing explants promotes cell division and facilitates exogenous gene integration, thereby improving transformation efficiency [29]. In this study, hypocotyls pre-cultured for 2 days showed optimal transformation efficiency when infected with *Agrobacterium* at an OD_600_ of 0.5. Lower or higher OD_600_ values reduced efficiency, likely because insufficient bacterial density limits infection, while excessive density increases explant mortality. The addition of acetosyringone (AS), the most widely used phenolic compound [30], further enhanced transformation. Testing three AS concentrations (100, 150, and 200 μmol/L) at infection times of 10, 15, and 20 min revealed that 200 μmol/L AS combined with a 15-min infection time yielded the highest transformation rate. A major challenge for stable transformation in *H. gyantsensis* is its poor rooting ability after *Agrobacterium* infection. Treating positive plantlets with 100 mg/L ABT1 for 40 min achieved a maximum rooting rate of only 12.5%, resulting in a final yield of approximately 4–5%. This rate is considerably lower than that reported for other species [31], indicating that further optimization of the rooting induction system is required to ensure the reliability of the transformation pipeline.

In this study, based on the established tissue culture regeneration system of *H. gyantsensis* [10], we systematically optimized key transformation parameters, including *Agrobacterium* suspension concentration, acetosyringone concentration, and infiltration time. By integrating technical experience from genetic transformation in woody plant models, we successfully established a stable genetic transformation system for *H. gyantsensis* for the first time. This system fills a technical gap in genetic engineering research of the genus *Hippophae* L., providing a robust platform for functional genomics and molecular breeding in *H. gyantsensis*.

In the breeding of *H. gyantsensis*, fruit size is a key trait for increasing yield and thus holds significant breeding value. However, *H. gyantsensis* suffers from issues such as a long fruiting cycle (taking 3 to 5 years to flower and 10 to 15 years to reach peak fruit production), small fruit size, and numerous thorns on the branches [32]. These factors severely hinder large-scale cultivation and mechanized harvesting, creating an urgent need to develop superior varieties with larger fruit and higher yields. Therefore, we are focusing on genes associated with the regulation of fruit size in *H. gyantsensis*. *fw2.2* and *fw3.2* are the two genes first reported to regulate fruit size, Cong et al. [33] overexpressed the *fw2.2* gene in transgenic tomato plants and demonstrated that *fw2.2* regulates the number of carpel cells in tomato fruits, leading to significantly smaller transgenic fruit size. In tomato, *fw3.2* encodes the cytochrome P450 (CYP450) protein SlKLUH [34], which arises from a single tandem duplication event (*fw3.2* ^dup^) and regulates tomato fruit weight in a dose-dependent manner. The *CYP78A* subfamily, a plant-specific clade of the CYP450 superfamily, has been extensively studied in *Arabidopsis thaliana*. In this model plant, *CYP78A* genes play pivotal roles in growth and development processes, including the regulation of flowering time and organ size [35].

A total of 14 *HgFWL* genes were identified in this study, and seven *CYP78A* family members were found in *H. gyantsensis*. Compared with *CYP78A* families in *A. thaliana*, tomato, and eggplant, the *HgCYP78A* family is relatively small in scale. *HgFWL2* (*Hgfw2.2*) and *HgCYP78A5* (*Hgfw3.2*) exhibit broad expression profiles rather than strict fruit-specificity, biologically consistent with the fundamental functions of their orthologs. The *fw2.2* (Cell Number Regulator/FWL family) [36,37] and *fw3.2* (KLUH/CYP78A family) genes are fundamentally regulators of cell division and proliferation [38]. Because cell division is highly active in vegetative tissues such as root tips, developing stems, and young leaves, it is expected that these genes are broadly expressed across these organs (Figure 5). Their regulation of fruit size is a specific manifestation of their general role in controlling cell proliferation during organogenesis. Therefore, their broad expression does not preclude their critical role in fruit development; rather, it supports their identity as fundamental cell division regulators. Heterologous expression of *Hgfw2.2* and *Hgfw3.2* in tomato significantly decreased and increased fruit size, respectively, consistent with their regulatory roles in fruit development and with the hypothesis that they influence organ size through cell number modulation.

However, the lack of phenotypic data on tomato fruit traits—such as ripening time, seed number, and seed size—poses certain limitations. Preliminary observations suggest that seed development in *Hgfw2.2* overexpression lines may be delayed, but quantitative validation is needed to confirm this phenotype and its correlation with transgene expression. Systematic measurement of ripening time could help determine whether *Hgfw2.2* or *Hgfw3.2* affects the fruit ripening process in transgenic lines.

Additionally, this study has several limitations. *Hgfw3.2* was overexpressed in *Solanum lycopersicum* L. and *H. gyantsensis*, and the resulting transgenic events were observed and analyzed. Compared with empty vector control (EV-OE) plants, T_0_ transgenic tomatoes overexpressing *Hgfw3.2* showed no significant changes in overall organ size or plant morphology (Figure 7D). However, the small sample size per group may have limited the statistical power to detect potential differences, if any. Quantitative data on seed development and fruit ripening kinetics in the overexpression lines are lacking. While qualitative images suggested potential developmental changes, these observations did not reach statistical significance in replicate experiments. Future studies with larger populations and time-course sampling will be essential to precisely quantify the effects of *Hgfw2.2* and *Hgfw3.2* on these developmental processes.

Similarly, due to the long fruit development cycle of *H. gyantsensis*, fruit phenotypic data have not yet been obtained after transplanting. Whether the growth and fruit traits (e.g., weight and cell number) of transgenic *H. gyantsensis* differ from those of wild-type plants requires further long-term observation and validation. Furthermore, although standard PCR and stringent antibiotic selection strongly indicate successful transformation, to detect the transgene in regenerated plantlets, PCR primers were designed against internal regions of the T-DNA. While this approach confirms the presence of the transgene, it cannot definitively rule out false positives arising from residual *Agrobacterium* or unintegrated T-DNA. To minimize such false positives, we employed rigorous surface sterilization, multiple rounds of subculture on antibiotic-containing media (timentin and kanamycin), and included water and non-transformed plant DNA as negative controls in all PCR runs. Nevertheless, because internal primer-based detection alone cannot confirm stable genomic integration, samples testing positive by PCR will be further validated in subsequent work using Southern blotting [39,40] or specific PCR spanning the T-DNA borders (T-DNA/genomic flanking sequences), and determination of transgene copy number remains a necessary next step in our follow-up studies, once sufficient biomass is generated from these woody plants. In follow-up studies, we will perform thermal asymmetric interlaced (TAIL)-PCR to isolate T-DNA flanking sequences from at least three independent lines showing distinct expression patterns, thereby directly confirming genomic integration. Southern blot analysis will be conducted if larger amounts of genomic DNA can be obtained from T_0_ generations.

In summary, by overcoming tissue culture recalcitrance and successfully generating PCR-positive transgenic seedlings, we have addressed the most critical and challenging bottleneck in *H. gyantsensis* biotechnology. Although endogenous fruit phenotype data remain to be confirmed, heterologous validation in tomato serves as a reliable functional surrogate, and the transgenic *H. gyantsensis* seedlings provide strong evidence for the feasibility of this transformation system.

## 4. Materials and Methods

### 4.1. Plant Materials

Seeds of *Hippophae gyantsensis* Lian were collected from the Gyangtse region of Tibet and preserved in the laboratory. The Gyantse area in Tibet is located at 89.6° E longitude and 28.92° N latitude, with an average altitude of about 4000 m. This region features a plateau temperate semi-arid climate, with an average annual temperature of 4.5 °C, annual precipitation of 300 mm, abundant sunshine, large diurnal temperature differences, and infertile alkaline soil. Tomatoes (*Solanum lycopersicum* L.) were cultivated in a greenhouse under controlled conditions (temperature: 25 ± 2 °C, photoperiod: 12–16 h, relative humidity: 60–70%). After fruit ripening, seeds were harvested for subsequent experiments.

### 4.2. Establishment of Genetic Transformation of H. gyantsensis

The preserved *Agrobacterium* strain GV3101 (purchased from Qingke Biotechnology Company, Beijing, China) was activated. Cotyledons and hypocotyls were excised from 20-day-old sterile seedlings as explant materials: cotyledons were transversely incised two to three times, while hypocotyls were cut into 1–1.5 cm segments. The explants were placed on pre-culture medium for pre-cultivation. Infection and co-cultivation were performed using different concentrations of *Agrobacterium* and acetosyringone (AS, purchased from Macklin Biochemical Technology Co., Ltd., Shanghai, China), with varying infection durations. An orthogonal experimental design was adopted for combinatorial optimization of these parameters.

After co-cultivation, the explants were washed three times with liquid sterile medium and then three times with sterile water. They were subsequently placed on recovery medium and cultured for 10 days. To induce resistant calli, the recovered explants were transferred to selection medium. Two weeks later, the explants were moved to resistant shoot induction medium, which was renewed every two weeks. When the resistant shoots reached approximately 1 cm in length, they were transferred to proliferation selection medium for further growth. Through this multi-step antibiotic screening process, resistant shoots were eventually obtained for subsequent molecular analysis.

### 4.3. Identification of Transgenic Plants

Genomic DNA was extracted from leaves of wild-type and genetically transformed *H. gyantsensis* using the CTAB method. Prior to DNA extraction, the putative transgenic tissues were subjected to the following treatments: thorough rinsing with sterile water containing 200 mg/L Timentin(purchased from Beijing LABLEAD Trading Co., Ltd, Beijing, China), followed by brief surface sterilization with 75% ethanol for 30 s to remove all surface-attached or endophytic *Agrobacterium* cells. In addition, DNA was extracted from newly regenerated tissues that had been subcultured for multiple rounds on selection medium and were distant from the original infection sites. The *E. coli* competent cells used for constructing the pSAK277 plasmid vector were DH5α (purchased from Sangone Biotech, Shanghai, China), and the *Agrobacterium* competent cells were GV3101 used for all transformation experiments. PCR amplification was performed with primers (Figure S4) designed for PCR cloning on the pSAK277 vector (digested with EcoRI and HindIII). The pSAK277 plasmid was used as the positive control, while water and wild-type plant DNA served as the negative controls. All PCR amplicons were subjected to gel electrophoresis, followed by visualization and photography using a gel imaging system.

### 4.4. Rooting Culture of Transgenic Plants

Transformed shoots were excised and transplanted into nutrient soil, then covered with a transparent plastic cover to maintain a high-humidity environment [41]. Positive seedlings were treated with ABT1 Rooting Powder No.1, with two variables set: hormone concentration and soaking duration. Seedlings without hormone treatment served as the control (CK). An average of thirty seedlings were planted per group to evaluate the effects of different hormone treatments on the rooting of *H. gyantsensis* positive seedlings (Table S4).

### 4.5. Identification and Screening of Genes Controlling Fruit Size in H. gyantsensis

BLASTp searches and screening of potential *FWL* and *CYP78A* family genes using Hidden Markov Model (HMM) files were performed against *H. gyantsensis* genomic data with tomato *FWL* and *CYP78A* protein sequences as queries. The chromosomal localization of *FWL* and *CYP78A* genes in *H. gyantsensis* was analyzed using its genome annotation file (GFF3), and the results were visualized with TBtools-II v2.472 software [42,43].

Subcellular localization of *FWL* and *CYP78A* proteins was predicted using the online tool PlantmPLoc (http://www.csbio.sjtu.edu.cn/bioinf/plant/, accessed on 3 March 2025). Multiple sequence alignment of conserved domain amino acid sequences was conducted using the Muscle program in MEGA 11.0 software, followed by sequence editing and optimization with Jalview software. The identified *H. gyantsensis FWL* and *CYP78A* protein sequences were aligned with those from *Arabidopsis thaliana*, tomato, rice, and eggplant (http://47.92.172.28:12068/Eggplant/home/index, accessed on 12 May 2025) using the Muscle program in MEGA 11.0, and a maximum likelihood (ML) phylogenetic tree was constructed.

The 2000 bp upstream sequences of *FWL* and *CYP78A* genes were extracted from *H. gyantsensis* genomic data and submitted to the PlantCARE website (https://bioinformatics.psb.ugent.be/webtools/plantcare/html/, accessed on 27 June 2025) for cis-acting element prediction. The distribution of cis-acting elements in the promoter regions was visualized using the heatmap tool in TBtools-II v2.472 software [44].

### 4.6. Transformation of Hgfw2.2 and Hgfw3.2

Based on the whole-genome sequence of *H. gyantsensis* and sequence alignment, specific primers were designed for the target genes (primer Hgfw2.2 and Hgfw3.2, Table S1). The coding sequences of *Hgfw2.2* and *Hgfw3.2* were amplified from *H. gyantsensis* cDNA, and the overexpression vectors pSAK277-*Hgfw2.2* and pSAK277-*Hgfw3.2* were subsequently constructed.

Tomato (*Solanum lycopersicum* L.) seeds were used as explant sources. Seeds were sterilized by immersion in 75% ethanol for 45 s, rinsed three times with sterile water, treated with 5% sodium hypochlorite for 5 min, and then rinsed four times with sterile water. The sterilized seeds were placed on germination medium. Seven- to eight-day-old axenic seedlings were selected; their cotyledons were cut into approximately 0.5 cm^2^ segments, placed flat on medium with the abaxial side up, and pre-cultured in darkness for 1 day. All cultures were maintained at 25 ± 1 °C under a 16 h light/8 h dark photoperiod with a light intensity of 27–40 μmol·m^−2^·s^−1^.

The *Agrobacterium tumefaciens* suspension was centrifuged at 4026× *g* for 8 min. The pellet was resuspended in liquid MS medium to an OD_600_ of 0.5. The pre-cultured tomato explants (1 day) were immersed in the bacterial suspension for 5 min, blotted dry with sterile filter paper, placed on co-cultivation medium (abaxial side up), and incubated in the dark at 25 ± 1 °C for 2 days.

After co-cultivation, explants were rinsed once with sterile water containing 200 mg/L Timentin, followed by 3–5 washes with sterile water. They were then placed on callus induction medium (adaxial side up) and cultured under the conditions described above until effective calli formed. The calli were transferred to shoot differentiation medium. After shoots emerged, they were moved to the shoot growth medium. When resistant shoots reached 1.0–1.5 cm in length, they were excised and transferred to rooting medium for root induction. Once T_0_ plants developed well-established roots, they were removed, rinsed thoroughly with tap water, acclimatized hydroponically for 2 days, and transplanted into a mixed substrate (nutrient soil: vermiculite: perlite = 2:1:1) for growth in a greenhouse under natural light.

Tomato lines overexpressing *Hgfw2.2* and *Hgfw3.2* were obtained via *Agrobacterium*-mediated transformation, and their fruit phenotypes were analyzed. At the reproductive stage (approximately 45 days after sowing), genomic DNA was extracted from leaves and detected by PCR. Tomato seedling growth and fruit weight and size at maturity were recorded. Meanwhile, *Hgfw3.2* was also introduced into *H. gyantsensis* using the same *Agrobacterium*-mediated transformation method (primer Hgfw3.2-JD, Table S1).

### 4.7. Quantitative Real-Time PCR (qRT-PCR) Analysis

Quantitative real-time PCR (qRT-PCR) was performed to analyze the expression levels of the candidate genes. Gene-specific primers for qRT-PCR were designed using Primer Premier 5.0 software based on the target gene sequences. Primer specificity was evaluated by BLAST+2.17.0 alignment against the *H. gyantsensis* genome (primers q-*Hgfw2.2* and q-*Hgfw3.2*, Table S1). Total RNA was extracted from fruits, roots, stems, and leaves, and reverse transcribed to obtain cDNA. qRT-PCR was conducted using the cDNA as template, with HgActin as the internal reference gene for *H. gyantsensis*. Relative gene expression levels were calculated using the 2^−ΔΔCt^ method. The same protocol was used for qRT-PCR analysis in *Solanum lycopersicum* L., where cDNA was extracted from leaves of transgenic plants and SlUBI was used as the internal reference gene (Table S1).

### 4.8. Data Statistics and Analysis

Plant regeneration efficiency(%) is defined as the percentage of explants that successfully regenerated shoots, calculated as the number of regenerated shoots divided by the total number of explants, multiplied by 100; PCR-positive transformation efficiency(%) refers to the proportion of regenerated shoots that were confirmed as transgenic by PCR analysis, calculated as the number of PCR-positive plants divided by the total number of regenerated shoots, multiplied by 100; Transformation efficiency(%) is defined as the overall percentage of explants that produced PCR-positive transgenic plants, calculated as the number of PCR-positive plants divided by the total number of explants, multiplied by 100.

Plant regeneration efficiency (%) = (Number of regenerated shoots/Total number of explants) ×100%;

PCR-positive transformation efficiency (%) = (Number of PCR-positive plants/Number of regenerated shoots) ×100%;

Transformation efficiency (%) = (Number of PCR-positive plants/Total number of explants) ×100%;

Rooting efficiency (%) = (Number of roots/Number of explants) ×100%.

## 5. Conclusions

In this study, an efficient *Agrobacterium*-mediated genetic transformation system for *H. gyantsensis* was established using hypocotyls as explants. The optimal kanamycin screening concentration was 20 mg/L. The highest transformation efficiency (36.67%) was achieved under the following conditions: *Agrobacterium* suspension OD_600_ = 0.5, acetosyringone (AS) concentration = 200 μmol/L, and infiltration time = 15 min. Positive shoots were treated with 100 mg/L rooting powder for 40 min before transplantation into nutrient soil, yielding a rooting rate of 12.5%. Based on the actual yield of rooted seedlings, the overall success rate was approximately 4–5%. Comprehensive expression analysis identified 14 *HgFWL* genes and seven *HgCYP78A* family members in *H. gyantsensis*. *Hgfw2.2* and *Hgfw3.2* were successfully cloned and heterologously expressed in tomato. Heterologous expression of *Hgfw2.2* and *Hgfw3.2* in tomato decreased and increased fruit size, respectively, consistent with their regulatory roles in fruit development. Overexpression of *Hgfw3.2* in tomato increased fruit size, indicating that this gene positively regulates fruit size. So, given the positive regulatory effect of *Hgfw3.2*, this gene was further transformed into *H. gyantsensis*. These findings provide an important theoretical basis for elucidating the molecular mechanisms of fruit development in *H. gyantsensis* and lay a foundation for breeding large-fruited varieties. Overall, the establishment of this transformation system not only enables targeted improvement of agronomic traits—such as fruit size through the *fw2.2*/*fw3.2* genes—to meet economic demands but also provides a key molecular tool for future investigations of the genetic basis of high-altitude adaptability and ecological functions in *Hippophae gyantsensis*.

## Figures and Tables

**Table 1:** Rooting efficiency under different treatment conditions.

Treatment	Number of Explants	Number of Roots	Rooting Efficiency (%)
CK	30	2	6.67%
T1	30	1	3.23%
T2	32	4	12.50%
T3	30	3	10.00%
T4	30	3	10.00%

Note: Rooting efficiency (%) = (Number of roots/Number of explants) ×100%.

## Data Availability

The RNA-Seq of *Hippophae gyantsensis* data accession ID is PRJCA062540 (National Genomics Data Center, Beijing, China).

## Supplementary Materials

The following supporting information can be downloaded at: https://www.mdpi.com/article/10.3390/plants15111615/s1, Table S1: All primer sequences used for the PCR identification of transgenic lines and qRT-PCR analysis; Table S2: Experimental design for all treatments in the genetic transformation; Table S3: Analysis results for transformation rate and positive transformation rate; Table S4: Ex vitro rooting test treatment combination; Table S5: Location and sequence information of *HgFWL* gene family members; Table S6: Location and sequence information of HgCYP78A gene family members; Figure S1: Conserved domain map from multiple sequence alignment of FWL proteins in tomato and *H. gyantsensis;* Figure S2: Conserved domain map from multiple sequence alignment of CYP78A proteins in tomato and *H.gyantsensis*; Figure S3: Phyloanalysis trees of the *HgFWL* and *HgCYP78A* genes in *Solanum lycopersicum* L. and *H.gyantsensis*; Figure S4: Plasmid profile of pSAK277.
