# Supplementary material for: Establishment of a Genetic Transformation System for Hippophae gyantsensis and the Regulatory Role of Hgfw2.2 and Hgfw3.2 in Fruit Size
Source: Plants (Basel). 2026 May 25;15(11):1615. doi: 10.3390/plants15111615 (PMC13259120; doi:10.3390/plants15111615)
Supplement: Supplementary file 1 [file plants-15-01615-s001.zip › plants-4298883-supplementary.pdf]

Supplementary Materials:

Table S1: All primer sequences used for the PCR identification of transgenic lines and qRT-PCR analysis.

Primer ID	Forward amplification primer	Reverse amplification primer
Hgfw2.2	ATGTACCAAAATAGTAATTATGGCGAC	GCGAGTCATGCCTTCTTCAACC
Hgfw3.2	ATGAAAACCCACATTGATTGTGTT	GAAAGACGAGCTTAACATACGCC
q-*Hgfw2.2*	CCTGCATCGGACATGAAACCTC	GGTGGTGGTGGTGGCTGAG
q-*Hgfw3.2*	ATCCTGTTCGTCGCTTGGTT	CCTTTGGGTCCAGGAATGGT
SlUBI	GCCAAAGAAGATCAAGCACA	TCAGCATTAGGGCACTCCTT
HgActin	GCTGAGCGATGCAAGAATGGAA	GCAGACAGGATGAGCAAGGAGA
Hgfw3.2-JD	CACAAGAGGGTGGTGAAGTGG	GGCGGACTTGAAGAAGTCGT
pSAK277-JD	TGAAACTCTGGCTCACCGAC	GCGGTTTTTCGCTTCTTGGT
pSAK277-homology arm	ACTAGTGGATCCAAAGAATTC	AGAAGTACTCTCGAGAAGCTT

Table S2: Experimental design for all treatments in the genetic transformation.

Treatment	OD	AS	Time	A	B	C	D(%)	E(%)	F(%)
T1	0.3	100	10	29	15	8	53.33%	27.59%	51.72%
T2			15	31	7	7	100.00%	22.58%	22.58%
T3			20	31	6	6	100.00%	19.35%	19.35%
T4		150	10	28	6	4	66.67%	14.29%	21.43%
T5			15	30	5	5	100.00%	16.67%	16.67%
T6			20	31	7	7	100.00%	22.58%	22.58%
T7		200	10	32	10	8	80.00%	25.00%	31.25%
T8			15	29	7	7	100.00%	24.14%	24.14%
T9			20	30	4	2	50.00%	6.67%	13.33%
T10	0.5	100	10	29	15	7	46.67%	24.14%	51.72%
T11			15	30	6	4	66.67%	13.33%	20.00%
T12			20	31	12	3	25.00%	9.68%	38.71%
T13		150	10	28	12	8	66.67%	28.57%	42.86%
T14			15	31	9	8	88.89%	25.81%	29.03%
T15			20	32	7	4	57.14%	12.50%	21.88%
T16		200	10	29	11	4	36.36%	13.79%	37.93%
T17			15	30	16	11	68.75%	36.67%	53.33%
T18			20	32	14	8	57.14%	25.00%	43.75%
T19	0.7	100	10	31	10	5	50.00%	16.13%	32.26%
T20			15	29	10	5	50.00%	17.24%	34.48%
T21			20	29	10	5	50.00%	17.24%	34.48%
T22		150	10	23	4	3	75.00%	13.04%	17.39%
T23			15	28	11	8	72.73%	28.57%	39.29%
T24			20	31	7	6	85.71%	19.35%	22.58%
T25		200	10	32	9	7	77.78%	21.88%	28.13%
T26			15	28	10	9	90.00%	32.14%	35.71%

T27	20	29	12	4	33.33%	13.79%	41.38%

Note: (**A**):Total number of explants; (**B**):Number of regenerated shoots; (**C**):Number of PCR-positive plants; (**D**):Plant regeneration efficiency(%)=(Number of regenerated shoots / Total number of explants)×100%; (**E**):PCR-positive transformation efficiency(%)=(Number of PCR-positive plants / Number of regenerated shoots)×100%; (**F**):Transformation efficiency(%)=(Number of PCR-positive plants / Total number of explants)×100%.

Table S3: Analysis results for transformation rate and positive transformation rate.

	Transformation rate（%）				Positive transformation rate（%）		
**Treatment**	**A**	**B**	**C**	**Treatment**	**A**	**B**	**C**
K1	1.79	1.67	1.84	K1	7.5	5.42	5.52
K2	1.89	1.81	2.17	K2	5.13	7.13	7.37
K3	1.79	1.99	1.46	K3	5.85	5.93	5.58
k1	0.20	0.19	0.20	k1	0.83	0.6	0.61
k2	0.21	0.20	0.24	k2	0.57	0.79	0.82
k3	0.20	0.22	0.16	k3	0.65	0.66	0.62
R	0.01	0.04	0.08	R	0.26	0.19	0.21

Note: (**A**):Bacterial suspension concentration(OD_{600}); (**B**):AS concentration(μmol/L); (**C**): Infiltration time (μmol/L). For the three variables (bacterial suspension concentration, AS concentration, and infiltration time), K1, K2 and K3 correspond to the total transformation efficiency or positive efficiency in genetic transformation, respectively; k1, k2 and k3 are the respective mean values of transformation efficiency or positive efficiency; R stands for the range, where a higher R value signifies that the variation of this factor level has a more pronounced impact on the outcomes.

Table S4: Ex vitro rooting test treatment combination.

**Treatment**	**Hormone concentration**	**processing time**
CK	-	-
T1	50mg/L	40min
T2	100mg/L	40min
T3	200mg/L	40min
T4	1000mg/L	5s

Table S5: Location and sequence information of *HgFWL* gene family members.

**Gene name**	**Gene ID**	**Gene Position**	**CDS**	**exon**	**intron**
HgFWL1	evm.model.chr2.1065	chr2	567	4	3
HgFWL2	evm.model.chr2.891	chr2	588	4	3
HgFWL3	evm.model.chr3.2041	chr3	723	4	3
HgFWL4	evm.model.chr4.1288	chr4	588	3	2
HgFWL5	evm.model.chr4.3302	chr4	2526	14	13
HgFWL6	evm.model.chr6.1269	chr6	714	5	4
HgFWL7	evm.model.chr6.1754	chr6	411	4	3
HgFWL8	evm.model.chr6.2801	chr6	726	4	3
HgFWL9	evm.model.chr8.1524	chr8	1005	5	4

HgFWL10	evm.model.chr10.1004	chr10	720	3	2
HgFWL11	evm.model.chr10.1082	chr10	1266	7	6
HgFWL12	evm.model.chr11.21	chr11	590	4	3
HgFWL13	evm.model.chr11.2343	chr11	441	4	3
HgFWL14	evm.model.chr12.931	chr12	738	5	4

Table S6: Location and sequence information of HgCYP78A gene family members.

**Gene name**	**Gene ID**	**Gene Position**	**CDS**	**exon**	**intron**
HgCYP78A1	evm.model.chr1.567	chr1	1299	2	1
HgCYP78A2	evm.model.chr3.1685	chr3	1611	2	1
HgCYP78A3	evm.model.chr4.2431	chr4	1650	3	2
HgCYP78A4	evm.model.chr7.208	chr7	1599	2	1
HgCYP78A5	evm.model.chr8.383	chr8	1656	3	2
HgCYP78A6	evm.model.chr11.266	chr11	1626	2	1
HgCYP78A7	evm.model.chr12.1006	chr12	1368	2	1

Figure S1: Conserved domain map from multiple sequence alignment of FWL proteins in tomato and *H. gyantsensis.*

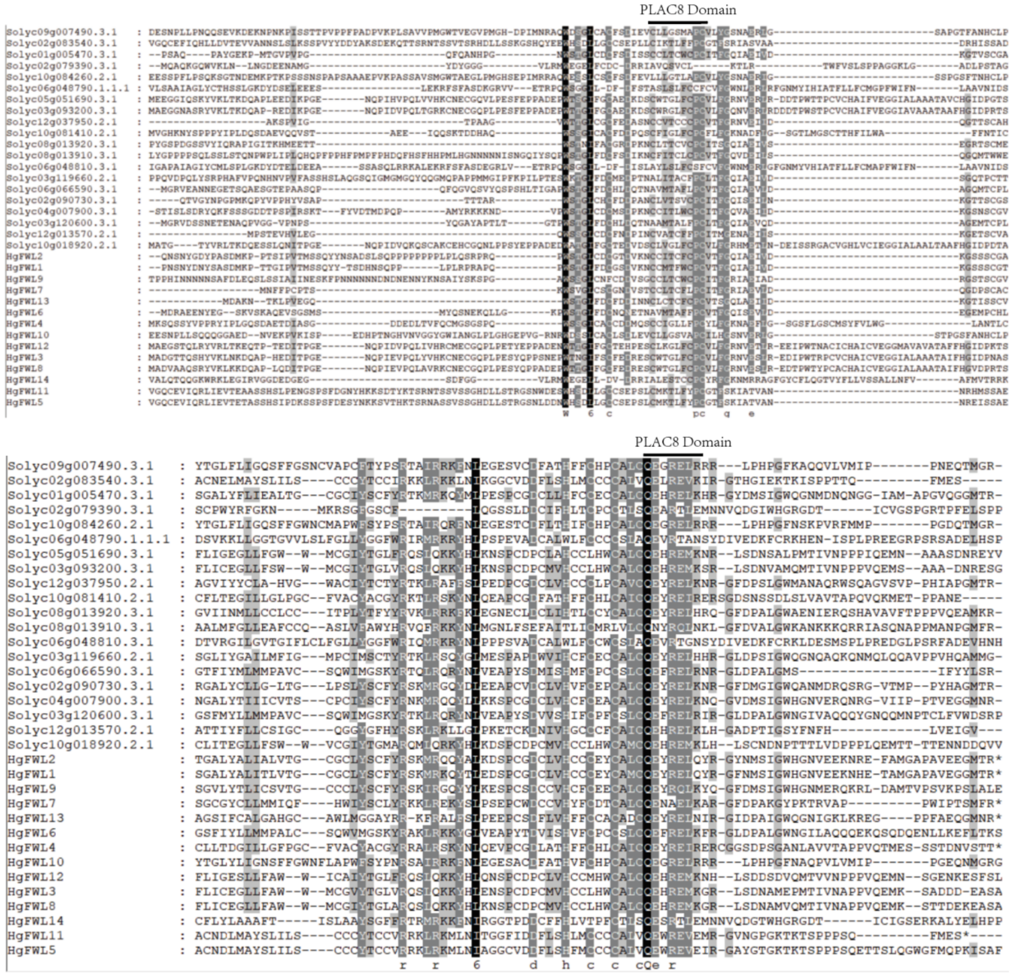

Figure S2: Conserved domain map from multiple sequence alignment of CYP78A proteins in tomato and *H.gyantsensis.*

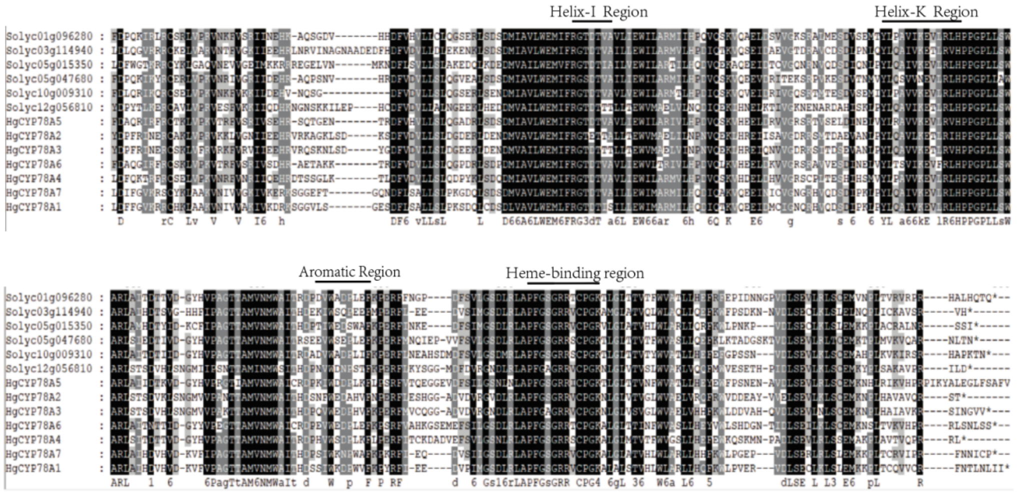

Figure S3: Phyloanalysis trees of the *HgFWL* and *HgCYP78A* genes in *Solanum lycopersicum* L. and *H.gyantsensis*.

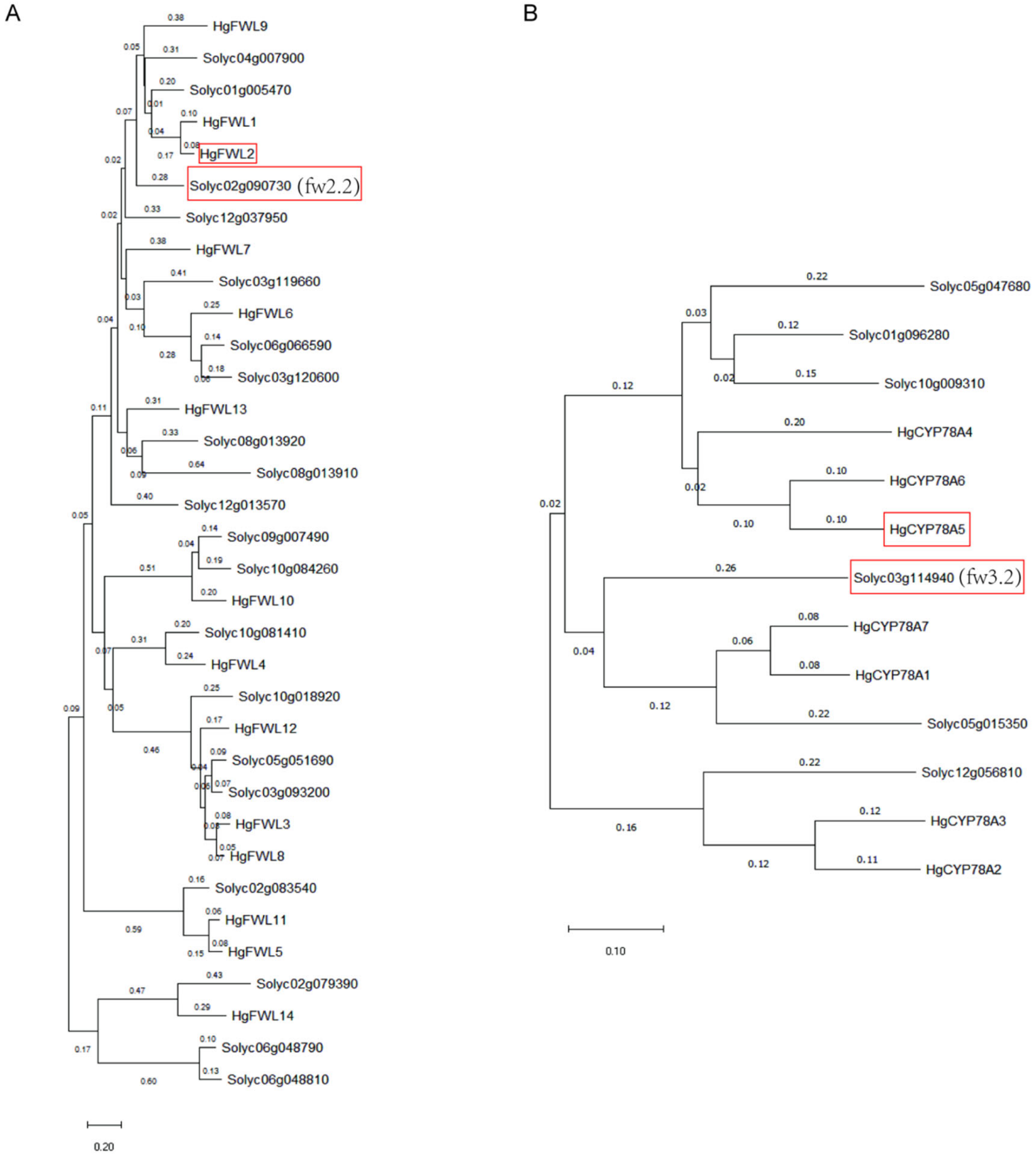

Note: (**A**):Phyloanalysis trees of the *HgFWL* genes in *Solanum lycopersicum* L. and *H.gyantsensis;* (**B**):Phyloanalysis trees of the *HgCYP78A* genes in *Solanum lycopersicum* L. and *H.gyantsensis.*

Figure S4: Plasmid profile of pSAK277.

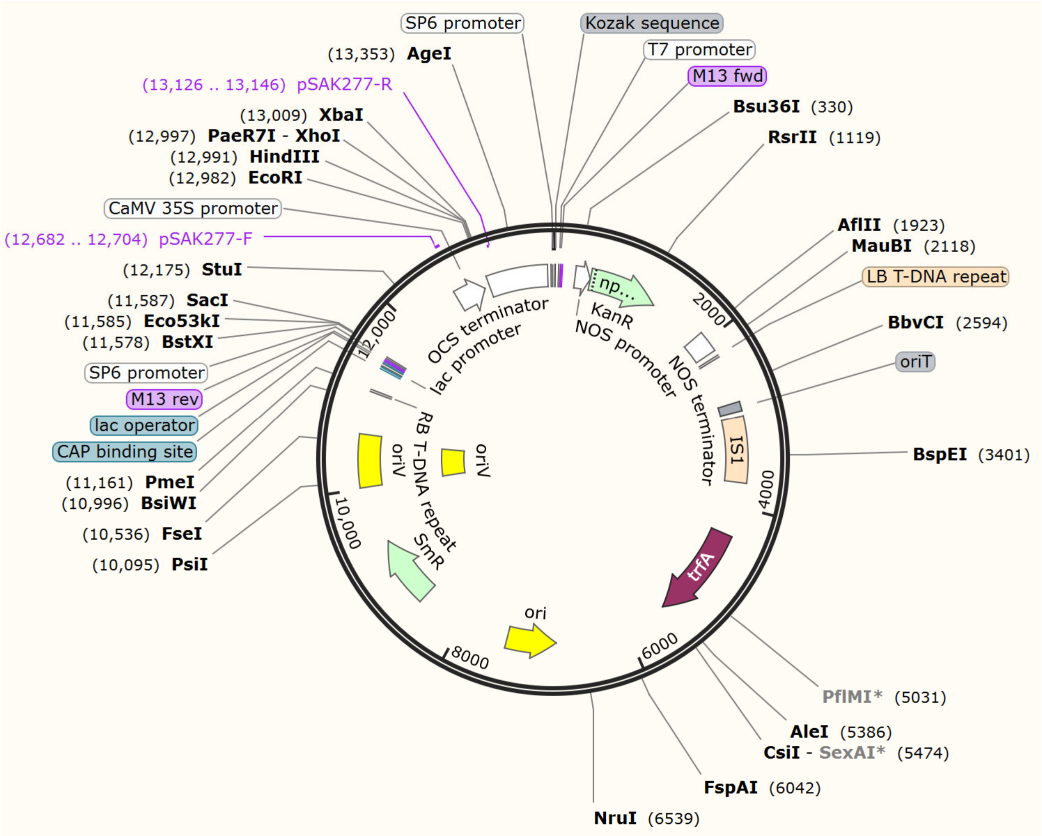

Note: Vector were digested with EcoRI and HindIII.
